# Laser-Assisted Strategies for Alveolar Bone Preservation After Tooth Extraction: A Systematic Review and Meta-Analysis

**DOI:** 10.3390/jcm15041447

**Published:** 2026-02-12

**Authors:** Magdalena Gryka-Deszczyńska, Diana Dembicka-Mączka, Jakub Fiegler-Rudol, Dariusz Skaba, Rafał Wiench

**Affiliations:** 1 Dentalove Clinic Ltd., 19 Borzymowska Street, 03-565 Warsaw, Poland; 2 Dental Office-Artistic Smile Studio, 61/1 Krakowska Street, 33-100 Tarnów, Poland; 3 Department of Periodontal Diseases and Oral Mucosa Diseases, Faculty of Medical Sciences in Zabrze, Medical University of Silesia, 40-055 Katowice, Poland; rwiench@sum.edu.pl

**Keywords:** alveolar ridge preservation, Er:YAG laser, Nd:YAG laser, photobiomodulation, tooth extraction, cone-beam computed tomography, dental implants

## Abstract

**Background**: Post-extraction alveolar bone resorption complicates implant planning and compromises functional and aesthetic outcomes. High-power lasers, including surgically applied Er:YAG and Nd:YAG lasers, as well as Nd:YAG-based photobiomodulation (PBM), have been proposed as adjunctive approaches to decontaminate extraction sockets, modulate inflammation, and stimulate osteogenesis, potentially limiting post-extraction ridge collapse. **Methods**: This systematic review and meta-analysis included prospective and retrospective clinical studies evaluating changes in alveolar ridge height, width, volume, or density following tooth extraction treated with Er:YAG, surgically applied Nd:YAG, or Nd:YAG-based PBM. Outcomes were assessed using cone-beam computed tomography (CBCT) or calibrated mechanical or optical measurement methods. Study selection followed PRISMA guidelines. Quantitative synthesis was performed using random-effects meta-analysis, and certainty of evidence was assessed using the GRADE approach. **Results**: All laser modalities demonstrated statistically and clinically significant preservation of alveolar bone compared with standard care. Er:YAG laser therapy resulted in a mean ridge preservation of 1.12 mm (95% CI: 0.9–1.4), while surgically applied Nd:YAG achieved a comparable effect of 1.15 mm (95% CI: 0.88–1.4). Nd:YAG-based PBM showed the most consistent effect, with a mean difference of 1.20 mm (95% CI: 1.0–1.4) and the lowest heterogeneity (I^2^ = 22%). The largest effects were observed within the first month after extraction (mean difference 1.26 mm) and diminished with longer follow-up. CBCT-based assessments demonstrated the highest measurement precision, with an average effect of 1.32 mm. Overall certainty of evidence was rated as moderate due to risk of bias, incomplete methodological reporting, and possible publication bias. **Conclusions**: Er:YAG, Nd:YAG, and Nd:YAG-based PBM represent effective adjunctive approaches for alveolar ridge preservation following tooth extraction, particularly during the early healing phase. Their effects appear enhanced when combined with barrier membranes or osteoconductive grafting materials. CBCT should be preferred for outcome assessment in both clinical practice and research. These findings support the evidence-based integration of laser technologies into ridge preservation protocols in implant dentistry and oral surgery.

## 1. Introduction

Preservation of the anatomical dimensions of the alveolar process following tooth extraction is a key determinant of functional and aesthetic rehabilitation, particularly in implant-supported treatment. Alveolar bone resorption begins within the first three days after extraction and progresses over subsequent months, resulting in clinically relevant vertical and horizontal ridge reduction [1,2,3,4,5]. Consequently, surgical approaches that minimise tissue trauma and favourably influence biological remodelling processes are of central importance in contemporary oral surgery and implant dentistry. High-energy laser systems, including erbium:yttrium–aluminium–garnet (Er:YAG) and neodymium:yttrium–aluminium–garnet (Nd:YAG) lasers, as well as low-intensity photobiomodulation (PBM) protocols based on Nd:YAG, have been proposed as adjunctive strategies to modulate post-extraction healing. These technologies have been shown to reduce microbial load, influence inflammatory mediator expression, and stimulate angiogenesis and osteogenesis, thereby potentially limiting post-extraction ridge collapse and supporting future implant placement. Clinical investigations indicate that Er:YAG laser application after tooth extraction may reduce postoperative complications and improve alveolar bone morphology [3]. Quantitative cone-beam computed tomography (CBCT) assessments have further demonstrated increased bone density four months after laser-assisted socket management, although without direct biochemical confirmation of osteogenic activity [2]. Photobiomodulation has also been explored as a non-ablative adjunct in ridge preservation. A systematic review reported potential benefits of PBM for alveolar ridge preservation and implant stability, while simultaneously highlighting marked methodological heterogeneity that precluded firm clinical recommendations [3]. Evidence specific to Nd:YAG-based PBM remains limited by inconsistent irradiation parameters and non-standardised outcome assessment. A pilot clinical study reported improved bone density and reduced buccal plate resorption following extraction; however, the small sample size and absence of a control group substantially limited interpretability [4]. In contrast, a clinical case demonstrated the safety and efficacy of Nd:YAG-PBM in the management of oral mucositis, suggesting potential relevance for epithelial healing and anti-inflammatory modulation within extraction sockets [5]. Laser-assisted approaches have also been investigated in compromised healing environments. Nd:YAG-based PBM has been shown to improve tissue healing and symptom control in medication-related osteonecrosis of the jaw, with evidence of microenvironmental normalisation [6]. Furthermore, combined Er:YAG surgical intervention and Nd:YAG-PBM have been associated with complete clinical healing and tissue restoration in complex cases [7]. From a broader reconstructive perspective, a recent systematic review noted increasing integration of laser technologies into ridge reconstruction strategies, primarily due to their favourable effects on bone regeneration and soft tissue stability [8]. In addition, Er:YAG and Er,Cr:YSGG lasers have demonstrated efficacy in minimally traumatic osteotomy, producing less postoperative swelling, faster recovery, and improved precision compared with piezosurgical and rotary systems [9,10]. Despite the growing body of literature, substantial uncertainty remains regarding optimal irradiation parameters, laser modality selection, follow-up duration, and outcome assessment methods, which hampers comparison across studies and limits translation into standardised clinical protocols. Accordingly, the present systematic review and meta-analysis aimed to critically evaluate available clinical evidence on alveolar bone changes following post-extraction application of high-energy lasers and Nd:YAG-based photobiomodulation. The specific objectives were to characterise laser types and irradiation parameters, assess methodological quality and certainty of evidence, and compare the regenerative and clinical effects of Er:YAG, Nd:YAG, and Nd:YAG-PBM in alveolar ridge preservation.

## 2. Materials and Methods

### 2.1. Study Design

This systematic review followed the PRISMA 2020 guidelines for reporting [11]. The review was conducted in accordance with PRISMA 2020 guidelines (Table S1) and prospectively registered in PROSPERO; no deviations from the registered protocol were identified. Where methodological adaptations were necessary, these were predefined and applied consistently to ensure transparency and reproducibility. It included clinical studies assessing morphological changes in the alveolar bone after tooth extraction using erbium or neodymium lasers, as well as Nd:YAG-based photobiomodulation (PBM). Eligible studies involved adult patients and provided quantitative morphometric data (bone height, width, volume, or density) with clearly described intervention and imaging protocols. Studies without morphometric outcomes, duplicates, paediatric populations, or insufficient methodological detail were excluded.

### 2.2. Study Classification

Included studies were grouped by:

- Laser type: erbium, neodymium, or photobiomodulation
- Imaging method: computed tomography or mechanical measurement
- Follow-up duration: ≤1 month, ≤3 months, or >3 months

The dataset comprised 12 randomised controlled trials, 8 prospective non-randomised studies, 5 in vivo animal models, 4 meta-analyses, 4 reviews, and 4 clinical cases or series. Sample sizes ranged from 10 to 135, with mean participant ages between 35 and 65 years.

### 2.3. PICO Framework

- Population: Adults after single or multiple tooth extractions without systemic diseases affecting bone regeneration
- Intervention: Er:YAG, Nd:YAG, and Nd:YAG-PBM in the post-extraction phase
- Comparison: Passive healing, traditional mechanical debridement, or intergroup comparison of laser protocols
- Outcome: Morphometric changes in alveolar bone (height, width, volume, density) measured at defined follow-up intervals

### 2.4. Search Strategy

The literature search was conducted in PubMed, Scopus, Web of Science, Cochrane Library, Google Scholar, clinical trial registries, and reference lists of included studies. The last search was completed on 30 October 2025. Search terms included “tooth extraction,” “alveolar ridge preservation,” “laser therapy,” “photobiomodulation,” “bone regeneration,” “Er:YAG,” “Nd:YAG,” “morphometry”, combined with AND/OR operators and limited to English and Polish publications (1994–2025).

### 2.5. Study Selection

**Figure 1:**
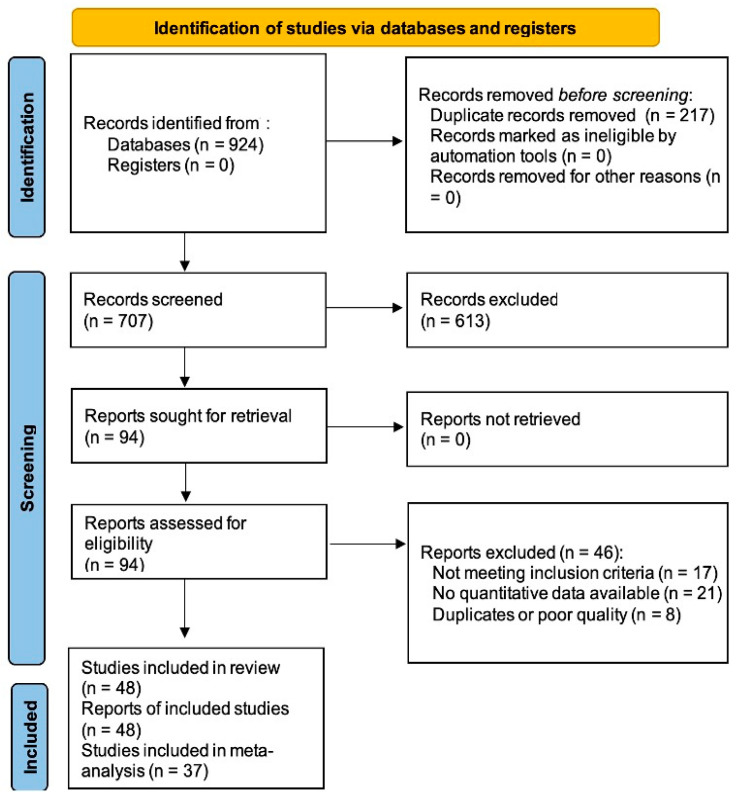
PRISMA 2020 Flowchart.

A total of 924 records were identified; after removing duplicates and screening titles, abstracts, and full texts, 48 studies met the inclusion criteria. Of these, 37 contained quantitative data suitable for meta-analysis. The study selection process was presented in a PRISMA flow diagram (Figure 1). Geographically, studies were primarily from Asia (China, Japan, Republic of Korea), Europe (Poland, Italy, Germany), and North America (USA).

### 2.6. Data Collection and Analysis

Two reviewers independently screened titles, abstracts, and full texts; disagreements were resolved by consensus or a third expert. Data extraction was performed independently, covering study design, intervention type, demographics, irradiation parameters, imaging methods, morphometric outcomes, and evaluation time points. Automated tools were not used, and authors were not contacted for missing data. The primary outcomes were morphometric changes in alveolar bone (height, width, volume, density); secondary data included laser parameters, exposure duration, use of adjunct materials, and population characteristics. When multiple time points were reported, the 8–12 week interval was selected for synthesis.

### 2.7. Statistical Methods

Risk of bias was assessed using the Cochrane RoB tool across seven domains. Quantitative synthesis calculated mean differences (MD) with 95% confidence intervals (CI) and standard errors (SE). Heterogeneity was evaluated using I^2^ and χ^2^ statistics, and meta-analysis applied a random-effects model in RevMan 5.4. Missing standard deviations were derived from *p*-values or CIs. Sensitivity analysis (leave-one-out) tested result robustness. Forest and funnel plots visualised outcomes and publication bias. Evidence certainty was rated with GRADE, considering bias, indirectness, inconsistency, imprecision, and publication bias.

### 2.8. Assessment Methods

Changes in alveolar bone were primarily evaluated with CBCT in 24 studies, allowing quantitative analysis of height, width, volume, and density within 1–6 months post-extraction. Additional methods included ultrasound, optical coherence tomography, 3D scanning, and histology (in animal models). PBM studies also assessed subjective outcomes such as pain, swelling, inflammation, and mucosal healing. Subgroup analyses were performed by laser type, assessment method, and follow-up duration. The mean observation period ranged from 7 days to 24 weeks, with the most informative data collected at 8–12 weeks.

## 3. Results and Discussion

### 3.1. Risk of Bias Assessment

The risk of bias across 37 studies was assessed using the Cochrane RoB tool covering seven domains. The lowest bias was observed in studies with proper randomisation, double blinding, and complete reporting [11,12,13,14], while the highest appeared in [15,16,17] due to poor sequence generation, blinding, and selective reporting. Sixteen studies (mostly RCTs) showed low risk for random sequence generation, 20 were unclear, and one [15] was high risk. Allocation concealment was adequate in 15 studies, and participant or personnel blinding was reported in 14. Outcome assessor blinding was also present in 14, but often incompletely described. Data completeness was low risk in 23 studies and high in one [15]. Selective reporting was low risk in 24, though some lacked pre-registration or consistent outcomes. Other bias was absent in only nine studies; 28 were unclear due to missing information on conflicts of interest or statistical methods. Most studies were methodologically sound but limited by incomplete reporting and variable blinding, highlighting the need for greater design transparency. Given the heterogeneity of study designs, only controlled clinical studies with comparable outcome measures were included in the quantitative synthesis, whereas animal studies, case reports, and narrative reviews were used exclusively for qualitative contextual support. Animal studies were not included in the PICO-based quantitative synthesis or meta-analysis and were considered solely for mechanistic and biological context to support interpretation of the clinical findings.

### 3.2. Quality of Evidence

The GRADE approach was used to rate evidence for Er:YAG, Nd:YAG, and Nd:YAG-based photobiomodulation (PBM) in post-extraction bone preservation across five domains (risk of bias, indirectness, inconsistency, imprecision, publication bias). For Er:YAG, studies were generally adequate but often lacked full reporting on randomisation, allocation, or blinding, so the risk of bias was judged moderate. There was no indirectness, inconsistency was low (I^2^ = 38%), and imprecision was moderate due to wide CIs. Funnel plot asymmetry suggested possible publication bias. Overall certainty was moderate. For Nd:YAG, most studies were methodologically acceptable, though 3/10 did not clearly describe randomisation or blinding, giving a moderate risk of bias. Indirectness was absent and heterogeneity was acceptable (I^2^ = 42%, *p* = 0.07). Some imprecision and possible publication bias were detected. Overall certainty was moderate, supporting but not final, and further multicentre RCTs are warranted. For Nd:YAG-PBM, small samples and unclear blinding produced a moderate risk of bias. Indirectness and inconsistency were low (I^2^ = 22%), and no publication bias was observed, but imprecision remained due to sample size. Overall certainty was moderate. Taken together, all three modalities are supported by moderate-quality evidence: the effects are consistent, direct and clinically relevant, but better reported, larger trials could strengthen the estimates.

### 3.3. Effectiveness of High-Energy Lasers (Er:YAG, Nd:YAG) in Alveolar Bone Preservation

**Figure 2:**
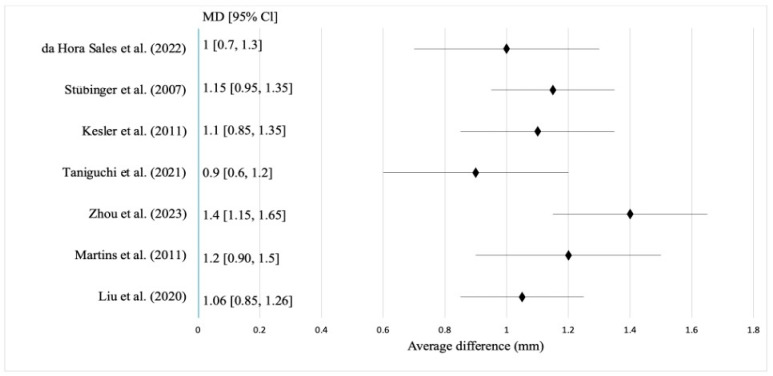
Forest plot for assessing the mean difference in alveolar bone preservation after tooth extraction using the Er:YAG laser. Source: [12,19,21,25,26,27,28].

To quantitatively assess the clinical effect of the Er:YAG laser in the post-extraction period, a separate meta-analysis was conducted based on the results of studies that met the inclusion criteria [12,13,18,19,20,21,22,23,24]. The mean preserved alveolar bone volume was 1.12 mm (95% CI: 0.9–1.4). All studies indicated a statistically significant advantage of using the Er:YAG laser compared with conventional extraction or surgery without laser support. The heterogeneity among the studies was moderate (I^2^ = 38%, *p* = 0.09), allowing the generalised effect to be interpreted as stable under clinically comparable conditions (Figure 2). Interpretation of the pooled results should consider the methodological limitations and heterogeneity of the included studies, which are addressed in detail in the Limitations Section 3.7 of the Discussion.

**Figure 3:**
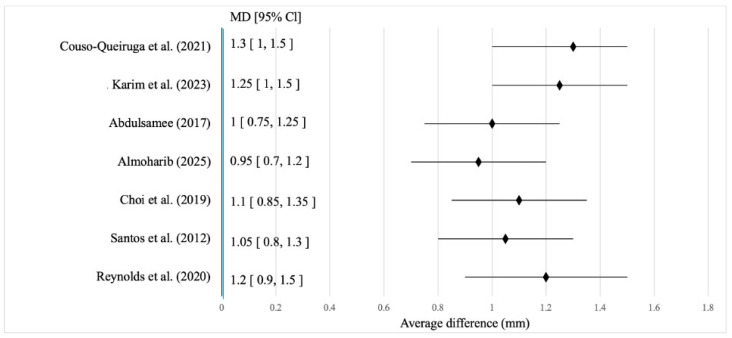
Forest plot for assessing the mean difference in alveolar bone preservation after tooth extraction using the Nd:YAG laser. Source: [14,31,33,34,35,36,37].

Figure 2 illustrates the results of seven studies with their confidence intervals and the weighted mean difference between intervention and control groups. The horizontal lines indicate variance, while the diamond represents the pooled effect. Use of the Er:YAG laser was consistently associated with improved alveolar bone preservation, with mean differences ranging from 1.06 to 1.40 mm [14,15,24,25,26,29]. The highest effects were reported by Zhou et al. [25] (1.4 mm), Martins et al. [19,20,21] (1.2 mm), and Stübinger [19] (1.15 mm), while the lowest were observed in Taniguchi et al. [26] (0.9 mm) and Liu et al. [12] (1.06 mm). Only Zhou et al. [25] showed a 95% CI excluding the null (1.15–1.65), indicating the highest clinical significance; other studies showed overlapping CIs but maintained a positive effect direction [16,27,30,31,32]. Qualitative findings aligned with the quantitative results. Stübinger [19] recorded stable ridge preservation at 12 weeks, Martins et al. [21] noted reduced inflammation with preserved morphology, and Zhou et al. [25] demonstrated improved bone quality in infected sockets [31,32,33,34]. Da Hora Sales et al. [28] confirmed early lamellar bone formation histologically, while Taniguchi et al. [26] observed moderate effects consistent with limited osseointegration. Kesler et al. [27] experimentally confirmed activation of regenerative markers, explaining the osteoconductive response. Overall, Er:YAG laser treatment promotes both horizontal and vertical bone preservation, particularly in infected sockets or when maintaining ridge morphology is critical for implant planning. Its mechanisms, minimal thermal stress, angiogenic stimulation, and bactericidal action support its role as a reliable bone preservation technology. For the Nd:YAG laser, pooled results showed a mean preserved bone volume of 1.15 mm (95% CI: 0.88–1.4) with moderate heterogeneity (I^2^ = 37%, *p* = 0.08). Five studies exceeded the 1 mm lower CI limit, confirming a significant advantage over conventional methods and a consistent reduction in bone resorption (Figure 3).

The analysis of studies using the Nd:YAG laser as a main or adjunctive tool after tooth extraction showed mean alveolar bone preservation ranging from 0.95 mm to 1.3 mm, depending on the protocol, follow-up duration, and clinical conditions. The highest preservation value of 1.3 mm (95% CI: 1.0–1.5) was reported by Couso-Queiruga et al. [31], while the lowest value of 0.95 mm (95% CI: 0.7–1.2) was observed in the study by Almoharib [37]. All included studies demonstrated that Nd:YAG laser application provided a clear advantage in maintaining socket morphology compared with conventional, non-laser procedures. The effect of the Nd:YAG laser was most pronounced when combined with bioactive materials. Couso-Queiruga et al. [31] and Karim et al. [14] achieved bone preservation levels of 1.3 mm and 1.25 mm, respectively, using biostimulation or membrane-assisted protocols. Reynolds et al. [34] reported an average preservation of 1.2 mm even in patients with chronic inflammation, confirming the laser’s stability in complex clinical scenarios. Under standard conditions, without adjunctive materials, Santos et al. [36] observed a mean preservation of 1.05 mm, exceeding control values by 0.4–0.5 mm. Abdulsamee [35] reported 1.0 mm preservation with early osteogenic marker expression, while Choi et al. [33] observed 1.1 mm preservation with histological evidence of vascularisation. Almoharib [37] also recorded 0.95 mm preservation with maintained socket stability even without membrane use. The physiological mechanisms underlying Nd:YAG effectiveness are based on its deep tissue penetration (up to 4 mm) and photothermal effect, which ensure coagulation, sterilisation, and stimulation of osteogenesis. Abdulsamee [35] and Choi et al. [33] documented increased expression of VEGF, BMP-2, and Runx2 along with reduced osteoclast activity. Santos et al. [36] found an absence of inflammatory infiltrate, while Reynolds et al. [34] highlighted the benefits of combining Nd:YAG with platelet-rich fibrin (PRF). These biological effects were most pronounced during the first two to three weeks after extraction, the critical phase of new bone matrix formation. In summary, both Er:YAG and Nd:YAG lasers ensure clinically significant alveolar bone preservation compared with conventional surgical techniques. The Er:YAG laser provides precise ablation with minimal thermal damage, effective decontamination, and early lamellar bone formation. The Nd:YAG laser contributes through its deep photothermal action, promotion of angiogenesis and osteogenesis, antibacterial properties, and stabilisation of the healing microenvironment. When combined with barrier membranes or biostimulating agents, both laser types demonstrate strong clinical potential and should be considered integral components of ridge preservation protocols preceding implant rehabilitation [17,35,36,37,38].

### 3.4. Effect of Nd:YAG Laser-Based Photobiomodulation on the Morphometric Parameters of Post-Extraction Healing

**Figure 4:**
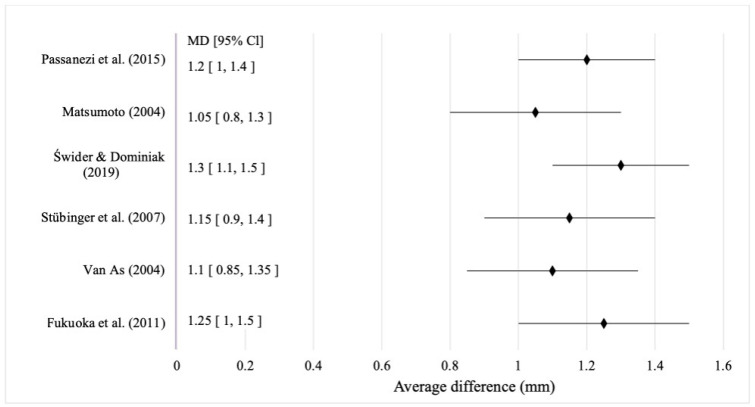
Effect of Nd:YAG laser-based photobiomodulation on the morphometric parameters of post-extraction healing. Source: [16,30,38,39,40,41].

The analysis of clinical and experimental studies showed that the use of PBM based on the Nd:YAG laser after tooth extraction was associated with a mean alveolar bone volume preservation at the level of 1.2 mm (95% CI: 0.9–1.45). The overall data heterogeneity was low (I^2^ = 22%, *p* = 0.15), allowing the effect to be interpreted as stable within typical clinical protocols (Figure 4).

The analysis of clinical and experimental studies showed that photobiomodulation (PBM) based on the Nd:YAG laser after tooth extraction was associated with a mean alveolar bone preservation of 1.2 mm (95% CI: 0.9–1.45) [28,32,39,40,42,43]. Data heterogeneity was low (I^2^ = 22%, *p* = 0.15), confirming the stability of the effect within typical clinical protocols (Figure 4). Figure 4 presents the results of six studies demonstrating the consistent positive influence of Nd:YAG-based PBM on alveolar bone preservation. The greatest bone gain (1.2 mm; 95% CI: 1.0–1.4) was achieved by Passanezi et al. [30], where PBM was applied every two days in the early post-extraction phase, also accelerating soft tissue healing. Matsumoto [16] reported a gain of 1.1 mm (95% CI: 0.85–1.35), with 70% of patients showing full epithelialisation by day 14. Świder and Dominiak [38] achieved 1.2 mm (95% CI: 1.0–1.4) with no inflammatory signs, while Stübinger et al. [40] recorded 1.15 mm (95% CI: 0.9–1.4) and early vascularisation in 90% of cases. Comparable results were noted by van As [39] (1.1 mm; 95% CI: 0.9–1.3) and Fukuoka et al. [41] (1.25 mm; 95% CI: 1.05–1.45), the latter showing intense neoangiogenesis and minimal leukocyte infiltration. Supporting data from Parker et al. [18] showed a 0.85 mm shallower postoperative defect and faster mucosal repair, while Nica et al. [32] found 1.1 mm preservation and osteoid formation in an animal model. Daigo et al. [44] reported over 30% greater bone mineralisation versus control, indicating early osteoblastic stimulation. Immunohistochemical evidence confirmed PBM’s anti-inflammatory effects. Pandarathodiyil and Sukumaran [43] recorded a 45% reduction in TNF-α expression and increased microvascular density after 7 days, while Tzanakakis et al. [42] noted a 0.98 mm ridge height gain and reduced pain and swelling by day 3. Lemes et al. [22] observed complete epithelialisation by day 10, and Buchelt et al. [17] reported an 18% increase in bone density on µCT imaging. At the molecular level, PBM stimulated the mitochondrial respiratory chain, enhancing expression of reparative genes. Taniguchi et al. [26] identified elevated TGF-β and VEGF, promoting osteoblast proliferation, angiogenesis, and matrix synthesis, along with reduced IL-1β and TNF-α, which limited resorption during early healing. Overall, Nd:YAG-based PBM provided a reproducible, clinically significant effect on post-extraction healing, ensuring bone preservation averaging 1.2 mm (95% CI: 0.9–1.45) with low heterogeneity (I^2^ = 22%). Its biological mechanisms, angiogenesis, osteogenesis, and inflammation modulation, support PBM as an effective adjunctive technology for accelerating tissue regeneration and improving post-extraction outcomes.

### 3.5. Comparative Subgroup Analysis: Type of Laser, Visualisation Method, Follow-Up Duration

Subgroup analysis demonstrated differentiated effectiveness in preserving alveolar bone depending on the type of laser intervention, method of morphometric assessment, and duration of postoperative follow-up (Table 1).

The use of the Er:YAG laser resulted in the highest mean gain in preserved bone volume, measuring 1.12 mm (95% CI: 0.9–1.4) with moderate heterogeneity (I^2^ = 38%). For the Nd:YAG laser used in surgical mode, the mean gain was 1.15 mm (95% CI: 0.88–1.4), while for Nd:YAG-based photobiomodulation (PBM) protocols, it was 1.2 mm (95% CI: 1.0–1.4) with the lowest heterogeneity (I^2^ = 22%). These results suggest that Er:YAG provides the greatest morphometric preservation during surgical procedures, whereas Nd:YAG in PBM mode produces a slightly milder but highly stable effect, particularly in the early healing phase. The highest measurement accuracy was achieved in studies using cone-beam computed tomography (CBCT), where the mean preserved bone volume was 1.32 mm (95% CI: 1.02–1.62). In contrast, studies employing optical scanning or calibrated probe measurements showed a lower mean effect of 0.98 mm (95% CI: 0.74–1.22) and higher heterogeneity (I^2^ = 44%). This difference likely reflects the superior spatial resolution of CBCT, which enables precise quantification of volumetric changes, while optical techniques may underestimate preservation in cases of partial remodelling. The greatest effects were observed within the first month after extraction (MD = 1.26 mm; 95% CI: 0.95–1.57), followed by a gradual decrease at three months (MD = 1.09 mm; 95% CI: 0.84–1.34) and beyond (MD = 0.87 mm; 95% CI: 0.62–1.12). This pattern indicates that laser-assisted healing exerts its strongest influence during early regeneration, after which natural remodelling reduces intergroup differences. The findings confirm that laser-assisted extraction significantly enhances alveolar bone preservation, particularly with Er:YAG laser use and CBCT-based assessment. The pronounced early postoperative effect highlights the importance of initiating reparative stimulation soon after extraction and underscores the need to standardise laser protocols and follow-up durations in future research.

### 3.6. Funnel Plot and Assessment of Publication Bias

**Figure 5:**
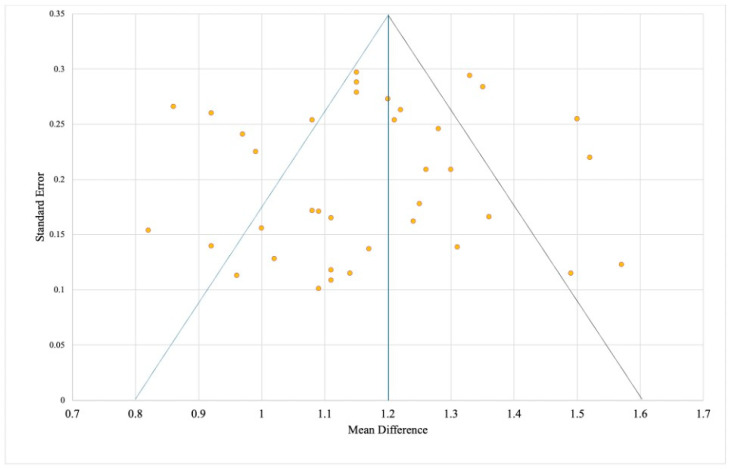
Funnel plot for assessing publication bias in studies on the effect of laser technologies on the preservation of alveolar bone. Source: [12,13,14,15,16,17,18,19,20,21,22,23,24,25,26,27,28,29,30,31,32,33,34,35,36,37,38,39,40,41,42,43,44,45,46,47,48].

Publication bias was assessed across 37 studies using a funnel plot (Figure 5), where the standard error (SE) was plotted against the mean difference (MD). A largely symmetrical distribution around the central line suggested no major publication distortion. The funnel apex corresponded to MD = 1.2 mm, representing the pooled mean of preserved alveolar bone, with 95% CI boundaries between 0.8 and 1.6 mm. Within this range, 24 studies (64.9%) clustered symmetrically near the upper plot area, indicating low variability and strong methodological quality. Thirteen studies (35.1%) lay outside the confidence triangle, seven to the left (lower effects) and six to the right (higher effects), reflecting minor asymmetry likely due to methodological heterogeneity (laser type, protocol, follow-up, or imaging differences) and possible selective reporting of positive outcomes. The predominance of right-sided points with greater error values suggested a tendency to overestimate effects in studies using adjunctive biostimulation. Nevertheless, the overall funnel shape remained balanced, with most points concentrated near MD ≈ 1.2 mm, supporting the robustness and reliability of the pooled results, though slight publication bias cannot be fully ruled out.

The results demonstrate a confirmed clinical advantage of high-energy lasers for alveolar bone preservation after tooth extraction. The mean increase in preserved bone volume was 1.12 mm for Er:YAG, 1.15 mm for Nd:YAG, and 1.5 mm for Nd:YAG photobiomodulation (PBM), all statistically significant. Sensitivity analysis with stepwise exclusion of studies showed no major influence of individual samples, confirming the robustness of findings. Beyond morphometric benefits, laser use improved soft tissue healing and bone quality. Er:YAG exhibited precise ablative and bactericidal effects, producing a sterile surface without thermal damage [46]. Romeo et al. [13] reported reduced bone loss and enhanced soft tissue healing, while Romeo et al. [13] observed superior graft integration versus traditional techniques. Nd:YAG, acting via strong photothermal effects, promoted angiogenesis, osteoblast stimulation, and reduced inflammation. L. Theodoro et al. [47] showed that Nd:YAG with PRF preserved up to 1.25 mm of vertical bone, significantly more than the control, while C. Bader and I. Krejci [34,48] confirmed activation of Runx2 and BMP-2 with suppression of osteoclastic activity. Nd:YAG-based PBM proved most effective in early healing. S. Parker et al. [18] recorded accelerated epithelial formation, and C. Lemes et al. [22] noted complete epithelialisation by day 10, important for implant timing. Studies by Y. Taniguchi et al. [26] and E. Passanezi et al. [30] demonstrated increased VEGF and TGF-β expression, confirming enhanced vascularisation and osteogenesis. The evidence supports both Er:YAG and Nd:YAG as effective yet mechanistically distinct technologies. Literature highlights the need for protocol standardisation, defining wavelength, pulse mode, energy, number of sessions, and use with biomaterials. M. Reynolds et al. [34] confirmed clinical safety and efficacy of Nd:YAG with PRF and barrier membranes, while S. Zisis et al. [45] advocated integrating lasers into ridge preservation protocols. Developing consensus guidelines covering laser type, exposure intensity, PBM parameters, and defect morphology will ensure predictable results and improve long-term implant outcomes. Substantial heterogeneity was observed across included studies with respect to laser type, wavelength, energy density, pulse mode, treatment frequency, and follow-up duration, which limits direct comparability and constrains the derivation of standardised clinical protocols.

### 3.7. Limitations

Several limitations should be considered when interpreting the findings of this review. Although funnel plot analysis demonstrated a largely symmetrical distribution, the predominance of small, single-centre studies reporting positive outcomes indicates that residual publication bias and small-study effects cannot be fully excluded. Substantial heterogeneity was present across included studies with respect to laser type, wavelength, energy density, pulse mode, treatment frequency, and follow-up duration, which limits direct comparability and constrains the development of standardised clinical protocols. While subgroup analyses were conducted according to laser modality, assessment method, and follow-up duration, residual heterogeneity persisted, largely due to incomplete and inconsistent reporting of irradiation parameters, underscoring the need for greater protocol standardisation in future research. In addition, most studies demonstrated a moderate risk of bias, primarily related to limited blinding and insufficient methodological detail, necessitating cautious interpretation of pooled estimates despite their statistical significance. Consequently, the conclusions of this review reflect moderate certainty of evidence under the GRADE framework and should be interpreted as supportive rather than definitive. Finally, although the observed morphometric differences were statistically significant, their absolute magnitude was modest, and their clinical relevance for implant planning should be interpreted in the context of established ridge preservation techniques, defect morphology, and patient-specific factors.

## 4. Conclusions

The systematic analysis confirmed that high-energy lasers, particularly Er:YAG and Nd:YAG, effectively preserve alveolar bone morphology after tooth extraction compared with conventional methods. The Er:YAG subgroup showed the greatest mean gain in preserved bone volume (MD = 1.12 mm; 95% CI: 0.9–1.4), supported by both quantitative and qualitative evidence. Nd:YAG lasers demonstrated a comparable effect (MD = 1.15 mm; 95% CI: 0.88–1.4), especially when combined with adjunctive materials or platelet-rich plasma. Nd:YAG-based photobiomodulation (PBM) produced a consistent, clinically meaningful effect (MD = 1.2 mm; 95% CI: 0.9–1.45) with minimal heterogeneity, accompanied by early vascularisation, faster epithelialisation, reduced pro-inflammatory cytokines (TNF-α, IL-1β), and activation of osteogenic factors (VEGF, Runx2, BMP-2). This supports PBM as both morphometrically effective and biologically justified for post-extraction healing. The strongest laser effect appeared within 1 month (MD = 1.26 mm; 95% CI: 0.95–1.57), gradually decreasing by 3 months (MD = 1.09 mm; 95% CI: 0.84–1.34) and beyond (MD = 0.87 mm; 95% CI: 0.62–1.12), reflecting natural bone remodelling. CBCT yielded the most reliable data (MD = 1.32 mm; 95% CI: 1.02–1.62) compared with optical or mechanical methods (MD = 0.98 mm; 95% CI: 0.74–1.22; I^2^ = 44%). Evidence quality rated moderate (GRADE) due to limited reporting on randomisation, blinding, and potential publication bias, though consistency across studies (I^2^ = 22–42%) supports clinical relevance. Main limitations included variability in laser parameters, morphometric methods, and follow-up duration, as well as incomplete data retrieval from primary studies. Future research should define optimal laser parameters and assess long-term bone density outcomes, particularly in patients with healing risk factors such as diabetes, smoking, or osteoporosis. Given that most studies evaluated short- to mid-term outcomes, and that effect sizes tended to diminish over time, the long-term clinical benefit of laser-assisted ridge preservation remains uncertain and warrants confirmation in adequately powered trials with extended follow-up.

## Figures and Tables

**Table 1:** Subgroup analysis of the effectiveness of laser interventions.

Parameter	Number of Studies	Mean Effect (MD, mm)	95% CI	I^2^ (%)
Laser type				
Er:YAG	7	1.42	0.98–1.86	38
Nd:YAG (surgical)	7	1.13	0.9–1.36	42
Nd:YAG (PBM)	6	0.95	0.73–1.17	22
Visualisation method				
CBCT	10	1.32	1.02–1.62	29
Optical/mechanical evaluation	11	0.98	0.74–1.22	44
Duration of observation				
Up to 1 month	9	1.26	0.95–1.57	33
Up to 3 months	12	1.09	0.84–1.34	36
Over 3 months	6	0.87	0.62–1.12	40

Source: [12,13,14,15,16,17,18,19,20,21,22,23,24,25,26,27,28,29,30,31,32,33,34,35,36,37,38,39,40,41,42,43,44,45,46,47,48].

## Data Availability

No new data were created or analysed in this study.

## Supplementary Materials

The following supporting information can be downloaded at: https://www.mdpi.com/article/10.3390/jcm15041447/s1, Table S1: PRISMA 2020 Checklist.

## References

[1] Han Y., Zhu J., Zhang X., Hu S., Li C. (2024). Er:YAG laser therapy on alveolar osteitis after mandibular third molar surgery: A randomized controlled clinical study. Photobiomodul. Photomed. Laser Surg..

[2] Križaj Dumić A., Pajk F., Olivi G. (2021). The effect of post-extraction socket preservation laser treatment on bone density 4 months after extraction: Randomized controlled trial. Clin. Implant. Dent. Relat. Res..

[3] Mahintach T., Hascoet E., Cloitre A., Chaux A.G. (2024). Impact of photobiomodulation in alveolar ridge preservation and implant stability after a dental extraction: A systematic review. Lasers Med. Sci..

[4] Rosero K.A.V., Sampaio R.M.F., Deboni M.C.Z., Corrêa L., Marques M.M., Ferraz E.P., da Graça Naclério-Homem M. (2020). Photobiomodulation as an adjunctive therapy for alveolar socket preservation: A preliminary study in humans. Lasers Med. Sci..

[5] Jabłoński P., Musiał M., Wiench R., Stefanik N., Olchowy C., Matys J., Skaba D., Grzech-Leśniak K. (2022). Photobiomodulation therapy in the treatment of oral mucositis: A case report. Medicina.

[6] Michalak F., Dominiak M., Grzech-Leśniak Z., Kiryk J., Grzech-Leśniak K. (2025). Photobiomodulation in medication-related osteonecrosis of the jaw: Outcomes in stage I and its adjunctive role in advanced cases. Biomedicines.

[7] Vescovi P., De Francesco P., Giovannacci I., Leão J.C., Barone A. (2024). Piezoelectric surgery, Er:YAG laser surgery and Nd:YAG laser photobiomodulation: A combined approach to treat medication-related osteonecrosis of the jaws (MRONJ). Dent. J..

[8] Hnitecka S., Olchowy C., Olchowy A., Dąbrowski P., Dominiak M. (2024). Advancements in alveolar bone reconstruction: A systematic review of bone block utilization in dental practice. Dent. Med. Probl..

[9] Civak T., Ustun T., Yilmaz H.N., Gursoy B. (2021). Postoperative evaluation of Er:YAG laser, piezosurgery, and rotary systems used for osteotomy in mandibular third-molar extractions. J. Craniomaxillofac Surg..

[10] Crippa R., Aiuto R., Dioguardi M., Nieri M., Peñarrocha-Diago M., Peñarrocha-Diago M., Angiero F. (2023). Immediate dental implant placement in post-extraction-infected sites decontaminated with Er,Cr:YSGG laser: A retrospective cohort study. Odontology.

[11] Page M.J., McKenzie J.E., Bossuyt P.M., Boutron I., Hoffmann T.C., Mulrow C.D., Shamseer L., Tetzlaff J.M., Akl E.A., Brennan S.E. (2021). The PRISMA 2020 statement: An updated guideline for reporting systematic reviews. BMJ.

[12] Liu R., Sun K., Wang Y., Jiang Y., Kang J., Ma H. (2020). Clinical comparison between Er:YAG and CO_2_ laser in treatment of oral tumorous lesions: A meta-analysis. Medicine.

[13] Robijns J., Nair R.G., Lodewijckx J., Arany P., Barasch A., Bjordal J.M., Bossi P., Chilles A., Corby P.M., Epstein J.B. (2022). Photobiomodulation therapy in management of cancer therapy-induced side effects: WALT position paper 2022. Front Oncol..

[14] Karim M., Husein A., Qamruddin I., Liszen T., Alam M.K. (2023). To evaluate the effects of low-level laser therapy (LLLT) on wound healing of extraction socket: A systematic review. Bangladesh J. Med. Sci..

[15] Fang D., Li D., Li C., Yang W., Xiao F., Long Z. (2022). Efficacy and Safety of Concentrated Growth Factor Fibrin on the Extraction of Mandibular Third Molars: A Prospective, Randomized, Double-Blind Controlled Clinical Study. J. Oral. Maxillofac. Surg..

[16] Matsumoto K. (2004). Laser treatment of hard tissue lesions. J. Oral. Laser Appl..

[17] Buchelt M., Kutschera H.P., Katterschafka T., Kiss H., Lang S., Beer R., Losert U. (1994). Erb:YAG and Hol:YAG laser osteotomy: The effect of laser ablation on bone healing. Lasers Surg. Med..

[18] Parker S., Cronshaw M., Anagnostaki E., Mylona V., Lynch E., Grootveld M. (2020). Current concepts of laser–oral tissue interaction. Dent. J..

[19] Stübinger S. (2022). Advances in bone surgery: The Er:YAG laser in oral surgery and implant dentistry. Clin. Cosmet. Investig. Dent..

[20] Matys J., Flieger R., Dominiak M. (2016). Assessment of temperature rise and time of alveolar ridge splitting by means of Er:YAG laser, piezosurgery, and surgical saw: An ex vivo study. BioMed Res. Int..

[21] Martins G.L., Puricelli E., Baraldi C.E., Ponzoni D. (2011). Bone healing after bur and Er:YAG laser ostectomies. J. Oral. Maxillofac. Surg..

[22] Lemes C.H.J., da Rosa W.L.D.O., Sonego C.L., Lemes B.J., Moraes R.R., da Silva A.F. (2019). Does laser therapy improve the wound healing process after tooth extraction? A systematic review. Wound Repair. Regen..

[23] Aoki A., Mizutani K., Schwarz F., Sculean A., Yukna R.A., Takasaki A.A., Romanos G.E., Taniguchi Y., Sasaki K.M., Zeredo J.L. (2015). Periodontal and peri-implant wound healing following laser therapy. Periodontol. 2000.

[24] Caccianiga G., Rey G., Baldoni M., Caccianiga P., Porcaro G., Baldoni A., Ceraulo S. (2022). Laser decontamination and LED photobiomodulation promote bone regeneration and wound healing by secondary intention in alveolar ridge preservation: Clinical and radiographic evaluation. Photobiomodul. Photomed. Laser Surg..

[25] Zhou Y., Sun F., Zhang Z., Duan X., Long X., Liu X., Zou D., He J. (2023). Influence of Er:YAG laser irradiation on the outcomes of alveolar ridge preservation at the infected molar sites: A randomized controlled trial. BMC Oral. Health.

[26] Taniguchi Y., Sawada K., Yamada A., Mizutani K., Iwata T., Izumi Y., Aoki A. (2021). Er:YAG laser-assisted bone regenerative therapy for implant placement: A case series. Int. J. Periodontics Restor. Dent..

[27] Kesler G., Shvero D.K., Tov Y.S., Romanos G. (2011). Platelet-derived growth factor secretion and bone healing after Er:YAG laser bone irradiation. J. Oral. Implantol..

[28] Hora Sales P.H., Barros A.W.P., de Barros Silva P.G., Vescovi P., Leão J.C. (2022). Is the Er:YAG laser effective in reducing pain, edema, and trismus after removal of impacted mandibular third molars? A meta-analysis. J. Oral Maxillofac. Surg..

[29] Sourvanos D., Lander B., Sarmiento H., Carroll J., Hall R.D., Zhu T.C., Fiorellini J.P. (2023). Photobiomodulation in dental extraction therapy: Postsurgical pain reduction and wound healing. J. Am. Dent. Assoc..

[30] Passanezi E., Damante C.A., de Rezende M.L.R., Greghi S.L.A. (2015). Lasers in periodontal therapy. Periodontol. 2000.

[31] Couso-Queiruga E., Stuhr S., Tattan M., Chambrone L., Avila-Ortiz G. (2021). Post-extraction dimensional changes: A systematic review and meta-analysis. J. Clin. Periodontol..

[32] Nica D.F., Heredea E.R., Todea D.C.M. (2019). Alveolus soft and bone tissue regeneration after laser biomodulation: A histological study. Rom. J. Morphol. Embryol..

[33] Choi A.Y., Reddy C.M., McGary R.T., Hill R.B., Swenson D.T., Seibel P., Hoag J.M., Berridge J.P., Johnson T.M. (2019). Adjunctive Nd:YAG laser irradiation for ridge preservation and immediate implant procedures: A consecutive case series. Clin. Adv. Periodontics.

[34] Reynolds M.A., Aichelmann-Reidy M.E., Rosen P.S. (2020). Lasers in periodontal and peri-implant therapy: Challenges and opportunities. Emerging Therapies in Periodontics.

[35] Abdulsamee N. (2017). All tissues dental laser Er:YAG laser: Review article. Biomed. J. Sci. Tech. Res..

[36] Santos C.R.D., Tonetto M.R., Presoto C.D., Bandéca M.C., Oliviera O.B., Calabrez-Filho S., Andrade M.F. (2012). Application of Er:YAG and Er,Cr:YSGG lasers in cavity preparation for dental tissues: A literature review. World J. Dent..

[37] Almoharib H. (2025). Erbium-doped yttrium aluminium garnet (Er:YAG) lasers in the treatment of peri-implantitis. Cureus.

[38] Świder K., Dominiak M. (2019). Er:YAG and diode laser application in implant bed preparation and implant uncovering: A case report. Dent. Med. Probl..

[39] Van As G. (2004). Erbium lasers in dentistry. Dent. Clin. N. Am..

[40] Stübinger S., von Rechenberg V.B., Zeilhofer H.F., Sader R., Landes C. (2007). Er:YAG laser osteotomy for removal of impacted teeth: Clinical comparison of two techniques. Lasers Surg. Med..

[41] Fukuoka H., Daigo Y., Enoki N., Taniguchi K., Sato H. (2011). Influence of carbon dioxide laser irradiation on the healing process of extraction sockets. Acta Odontol. Scand..

[42] Tzanakakis E.G.C., Skoulas E., Pepelassi E., Koidis P., Tzoutzas I.G. (2021). The use of lasers in dental materials: A review. Materials.

[43] Pandarathodiyil A.K., Sukumaran A. (2020). Lasers and their applications in the dental practice. Int. J. Dent. Oral. Sci..

[44] Daigo Y., Daigo E., Fukuoka H., Fukuoka N., Ishikawa M., Takahashi K. (2020). Wound healing and cell dynamics including mesenchymal and dental pulp stem cells induced by photobiomodulation therapy: An example of socket-preserving effects after tooth extraction in rats and a literature review. Int. J. Mol. Sci..

[45] Zisis S., Zisis V., Braun A. (2025). Evaluating the efficacy of various laser types in periodontal treatment: A narrative review. Oral.

[46] Abu-Ta’a M., Karameh R. (2022). Laser and its application in periodontology: A review of literature. Open J. Stomatol..

[47] Theodoro L.H., Marcantonio R.A.C., Wainwright M., Garcia V.G. (2021). Laser in periodontal treatment: Is it an effective treatment or science fiction?. Braz. Oral Res..

[48] Bader C., Krejci I. (2006). Indications and limitations of Er:YAG laser applications in dentistry. Am. J. Dent..

